# American Journal of Obstetrics

**Published:** 1868-05

**Authors:** 


AMERICAN JOURNAL OF OBSTETRICS.—The first number of the American Journal of Obstetrics has been received. It is edited by E. Nœggerath, M. D., and B. F. Dawson, M. D. It contains four original articles, editorial and miscellaneous articles, abstracts, etc. It is published by Moorhead, Bond & Co., New York. The first number leads us to predict a popular and valuable journal, though we cannot fully appreciate the occasion for a journal devoted wholly to obstetrics and the diseases of women and children; perhaps such division is desirable, but we have never yet seen how the various departments of medicine can be more profitably cultivated when separated from each other.

